# Incidence and mortality rates of selected infection-related cancers in Puerto Rico and in the United States

**DOI:** 10.1186/1750-9378-5-10

**Published:** 2010-05-14

**Authors:** Ana P Ortiz, Marievelisse Soto-Salgado, William A Calo, Guillermo Tortolero-Luna, Cynthia M Pérez, Carlos J Romero, Javier Pérez, Nayda Figueroa-Vallés, Erick Suárez

**Affiliations:** 1 Cancer Control and Population Sciences Program, University of Puerto Rico Comprehensive Cancer Center, San Juan, Puerto Rico; 2 Graduate School of Public Health, Department of Biostatistics and Epidemiology, Medical Sciences Campus, University of Puerto Rico, San Juan, Puerto Rico; 3 Medical Sciences Campus, Puerto Rico Cancer Center, University of Puerto Rico, San Juan, Puerto Rico; 4 School of Medicine, Gastroenterology Research Unit, Medical Sciences Campus, University of Puerto Rico, San Juan, Puerto Rico; 5 Puerto Rico Central Cancer Registry, San Juan, Puerto Rico

## Abstract

**Background:**

In 2002, 17.8% of the global cancer burden was attributable to infections. This study assessed the age-standardized incidence and mortality rates of stomach, liver, and cervical cancer in Puerto Rico (PR) for the period 1992-2003 and compared them to those of Hispanics (USH), non-Hispanic Whites (NHW), and non-Hispanic Blacks (NHB) in the United States (US).

**Methods:**

Age-standardized rates [ASR(World)] were calculated based on cancer incidence and mortality data from the PR Cancer Central Registry and SEER, using the direct method and the world population as the standard. Annual percent changes (APC) were calculated using the Poisson regression model from 1992-2003.

**Results:**

The incidence and mortality rates from stomach, liver and cervical cancer were lower in NHW than PR; with the exception of mortality from cervical cancer which was similar in both populations. Meanwhile, the incidence rates of stomach, liver and cervical cancers were similar between NHB and PR; except for NHB women who had a lower incidence rate of liver cancer than women in PR. NHB had a lower mortality from liver cancer than persons in PR, and similar mortality from stomach cancer.

**Conclusions:**

The burden of liver, stomach, and cervical cancer in PR compares to that of USH and NHB and continues to be a public health priority. Public health efforts are necessary to further decrease the burden of cancers associated to infections in these groups, the largest minority population groups in the US. Future studies need to identify factors that may prevent infections with cancer-related agents in these populations. Strategies to increase the use of preventive strategies, such as vaccination and screening, among minority populations should also be developed.

## Background

Infection with several viruses and bacteria has been associated to the development of various cancer types [1]. Nearly 17.8% of the global cancer burden is attributable to infectious agents [2], with a higher percentage in developing countries (26.3%) than in developed countries (7.7%) [3]. The principal infectious agents associated with cancer morbidity worldwide are *Helicobacter pylori *(H. pylori) (5.5% of all cancers), human papilloma viruses (HPV) (5.2% of all cancers), and hepatitis B (HBV) and hepatitis C viruses (HCV) (4.9% of all cancers); these agents account for 87.6% of the total cancer burden associated to infections [2]. More specifically, between 74%-78% of all stomach cancers worldwide have been attributed to infection with *H. pylori*, 85.4% of hepatocellular carcinoma is attributable to HBV (54.4%) or HCV (31.0%) infection [3], while persistent infection with certain types of HPV has been established as a necessary cause for cervical cancer, accounting for 100% of these tumors [2]. Moreover, HPV infection has been associated to cancer of the vulva, vagina, penis, anus, oral cavity and oropharynx [3]. Among other cancer types, bladder and nasopharyngeal cancer, as well as various types of lymphomas and sarcomas, have also been associated to other infectious agents such as *Schistosoma haematobium*, Epstein-Barr virus, HCV, *H. Pylori*, human T-cell leukemia virus type 1 (HTLV-1), and human herpes virus 8 (HHS-8) [1,3-5]. Nonetheless, the percent of all cancers worldwide associated to these previously mentioned infections is less than 3% [3].

**Figure 1:**
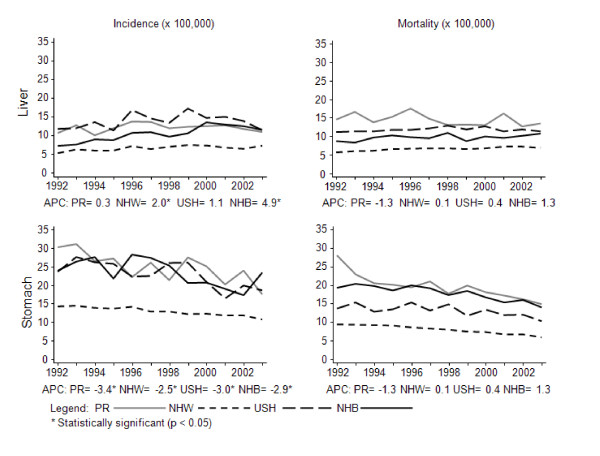
**Trends for stomach and liver cancer among men in all racial/ethnic groups, 1992-2003**.

Worldwide, among infection-related cancers, stomach, liver, and cervix uteri cancers not only account for the vast majority of the total cancer burden associated to infections [3,6], but they also have the highest incidence figures [1]. Although the incidence of total cancer in Puerto Rico (PR) is lower than that of the United States (US), the incidence from infection-related cancers such as stomach, liver and cervical cancer is higher in the island than in the US, particularly among non-Hispanic Whites (NHW). Contrary to the US, these cancer types also rank among the leading cancer sites in incidence and mortality in PR [7-12]. This pattern is similar to patterns observed among Hispanics in the US (USH) who show lower incidence and mortality rates from total cancer than NHW for the most frequent cancer types, although they experience a higher incidence of cancers related to infectious agents [13-15], similar to that of their countries of origin [16].

Information on the current burden of infection-related cancers in PR and how it compares with other US racial/ethnic groups is limited because these comparisons have mainly focused on USH, NHW and non-Hispanic Blacks (NHB) [14,15]. Understanding patterns of cancer incidence in diverse racial/ethnic groups is essential to further understand risk factors for disease occurrence in specific populations and to direct appropriate cancer prevention and control efforts for specific population sub-groups. Thus, this study aimed to assess the age-standardized incidence and mortality rates of stomach, liver and cervical cancer in PR and compared them to those of USH, NHW, and NHB in the US for the period 1992-2003.

## Methods

### Data sources

Incident cases and deaths for stomach, liver and cervical cancer for all racial/ethnic groups were obtained from the PR Central Cancer Registry (PRCCR) [17,18] and the Surveillance, Epidemiology and End Results Program (SEER) [19,20], respectively. The SEER program is a national cancer surveillance database that collects and reports incidence and survival data from a sample of the US population. The PRCCR is the fourth oldest population-based cancer registry in the world [21] and collects information on cancer in PR since 1951. The PRCCR is part of the National Program of Cancer Registries (NPCR) administered by the Centers of Disease Control and Prevention (CDC). The PRCCR uses the coding standards of the SEER and of the North American Association of Central Cancer Registries (NAACCR); thus, the registry is fully comparable with SEER data. In the year 2003, a CDC audit concluded that 95.3% of all cancer cases diagnosed or treated in hospital facilities in PR were appropriately reported to the PRCCR; a result comparable to the US median (95%) [22]. The third revision of the International Classification of Diseases for Oncology (ICD-O-3) was used to select all cases diagnosed from stomach, liver and cervical cancer between 2001 and 2003. Cases from 1992 to 2000, which were originally reported using ICD-O-2, were converted to ICD-O-3. The specific codes for the types of cancer studied were: cervical cancer (C53.0-C53.9), cancer of the liver and intraepithelial bile duct (C22.0-C22.1) and stomach cancer (C16.0-C16.9). Cancer mortality data for PR and the US (NHW, NHB, USH) was obtained, respectively, from the PRCCR as reported by death certificates enacted by vital statistics from the Puerto Rico Department of Health and from the SEER program as reported by the National Center for Health Statistics (NCHS). Causes of death were coded and classified according to the tenth edition International Classification of Diseases (ICD-10). Mortality codes for the specific types of cancer included: cervical cancer (ICD-9: 180.0-180.9, ICD-10: C53), cancer of the liver and intraepithelial bile duct (ICD-9:155.0-155.2, ICD-10:C22.0, C22.0-C22.4, C22.7, C22.9) and stomach cancer (ICD-9:151.0-151.9, ICD-10:C16).

### Statistical Analysis

For each racial/ethnic group, annual age-standardized incidence and mortality rates [ASR(World)] (per 100,000) of stomach, liver and cervical cancer for 1992-2003 were calculated, using the direct method (mortality rates were based on underling cause of death) and the world population as the standard [23]. To assess the trends in risk for specific sites, the annual ASR(World) were calculated by sex, as follows:  where w_j _is the proportion of persons in the *j*-th age group of the standard population, d^k^_ij _is the number of cases (new cases or deaths) in the *j*-th age group for the *i*-th ethnic group in the *k*-th year, and n^k^_ij _is the population in the *j*-th age group for the *i*-th ethnic group in the *k*-th year. The annual percent change (APC) of the ASR(World) was estimated using the joinpoint regression model [24]. The Joinpoint Regression Program version 3.3 [25] was used for the APC estimation using the following parameters: 1) logarithmic transformation of the rate, 2) zero joinpoint model, 3) Poisson model using rate, 4) uncorrelated error model, and 5) Hudson's method. To assess differences in the incidence and mortality rates of stomach, liver and cervical cancer between PR as compared to the other racial/ethnic groups, the ASR(World) were grouped from 1999 to 2003 (averaged over the 5-year period). Then, the standardized incidence ratio (SIR) and the standardized mortality ratio (SMR) were estimated with 95% confidence intervals [26], to compare the racial/ethnic risk with PR as the reference group. We also compared sex differences in each racial/ethnic groups, with females as the reference group, in the occurrence of stomach and liver cancer. The STATA System release 10.0 (STATA Corp, College Station, TX, USA) was used for the statistical analysis.

## Results

### Stomach cancer

#### Rates (1999-2003)

In men, the incidence rates (per 100,000) of stomach cancer ranged from 11.7 in NHW to 22.5 in PR; while in women rates ranged from 5.5 in NHW to 12.9 in NHB. NHW men and women had 48% (SIR = 0.52, 95% CI = 0.48, 0.57) and 50% (SIR = 0.50, 95% CI = 0.46, 0.55) lower risk, respectively, of stomach cancer, than their counterparts in PR (Table 1). No significant differences in stomach cancer risk were observed between USH and NHB men and women as compared to PR, except for NHB females who had a higher risk of the disease as compared to women in PR (SIR = 1.17, 95% CI = 1.03-1.33). The mortality rates (per 100,000) from stomach cancer in men ranged from 6.8 in NHW to 17.1 in PR, and from 3.5 in NHW to 8.7 in NHB in women (Table 1). NHW and USH men and women had a lower risk of death than their Puerto Rican counterparts (p < 0.05); whereas, no significant differences in mortality were observed between NHB and PR (p > 0.05; Table 1). In all racial/ethnic groups, the incidence and mortality rates of stomach cancer were significantly higher among men as compared to women (p < 0.05) (Table 1).

**Table 1:** ASR(World) for incidence and mortality (per 100,000) for stomach, liver and cervical cancer during 1998-2003.

	Age Standardized Rate (ASR)				Standardized Relative Ratio SRR (95% CI)		
	**PR**	**NHW**	**USH**	**NHB**	**NHW/PR**	**USH/PR**	**NHB/PR**

***Incidence***							

**Stomach**							

Male	22.53	11.71	20.13	20.22	0.52 (0.48-0.57)	0.89 (0.79-1.01)	0.90 (0.79-1.01)

Females	11.00	5.51	12.51	12.86	0.50 (0.46-0.55)	1.14 (1.00-1.29)	1.17 (1.03-1.33)

SRR M^a ^vs F^b^	2.05 (1.82-2.30)	2.13 (2.02-2.25)	1.61 (1.42-1.83)	1.57 (1.38-1.76)			

**Liver**							

Male	11.89	6.96	14.23	12.20	0.59 (0.53-0.65)	1.20 (1.05-1.37)	1.03 (0.89-1.18)

Females	5.94	2.95	6.40	4.21	0.50 (0.44-0.57)	1.08 (0.91-1.27)	0.71 (0.59-0.85)

SRR M^a ^vs F^b^	2.00 (1.72-2.33)	2.44 (2.27-2.61)	2.22 (1.92-2.59)	2.92 (2.46-3.27)			

**Cervix**							

Females	9.07	5.90	12.68	10.01	0.65 (0.60-0.70)	1.40 (1.28-1.53)	1.10 (0.99-1.23)

***Mortality***							

**Stomach**							

Male	17.07	6.75	11.76	16.01	0.40 (0.36-0.43)	0.69 (0.62-0.77)	0.94 (0.85-1.04)

Female	8.02	3.51	6.70	8.70	0.44 (0.39-0.49)	0.84 (0.74-0.94)	1.08 (0.97-1.22)

SRR M^a ^vs F^b^	2.13 (1.85-2.45)	1.92 (1.88-1.97)	1.76 (1.62-1.90)	1.84 (1.75-1.92)			

**Liver**							

Male	13.59	6.86	11.73	9.88	0.50 (0.46-0.55)	0.86 (0.78-0.96)	0.73 (0.66-0.80)

Females	7.82	3.31	6.46	4.68	0.42 (0.38-0.47)	0.83 (0.73-0.93)	0.60 (0.53-0.67)

SRR M^a ^vs F^b^	1.73 (1.51-1.99)	2.07 (2.03-2.12)	1.81 (1.68-1.96)	2.12 (1.99-2.22)			

**Cervix**							

Female	2.38	2.11	3.36	5.12	0.89 (0.76-1.04)	1.41 (1.20-1.68)	2.16 (1.84-2.55)

^a^Male

^b^Female

#### Trends

**Figure 2:**
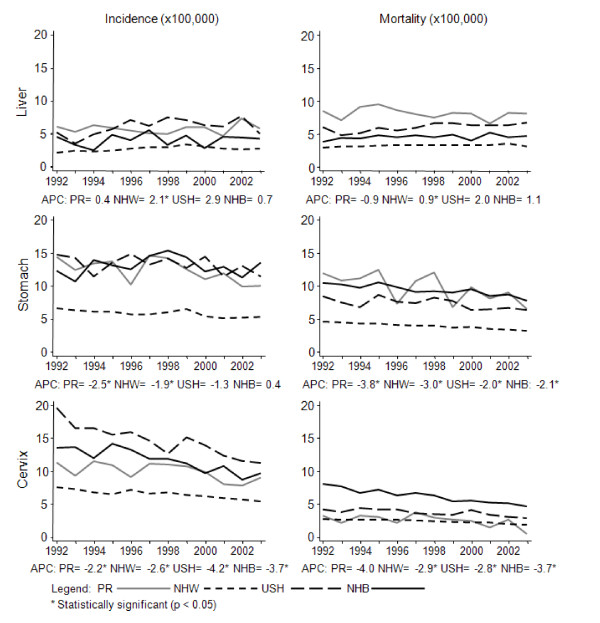
**Trends for stomach, liver and cervical cancer among women in all racial/ethnic groups, 1992-2003**.

From 1992-2003, the incidence of stomach cancer decreased 2%-3% per year in men and women from most racial/ethnic groups, except for USH and NHB women who showed relatively constant trends (Figure 1). Meanwhile, mortality showed a significantly decreasing trend for women from all racial/ethnic groups (APC between 2-4% for all groups) and a constant trend for men (Figure 2).

### Liver cancer

#### Rates (1999-2003)

The incidence rates (per 100,000) of liver cancer in men ranged from 7.0 in NHW to 14.2 in USH; and in women from 3.0 in NHW to 6.4 in USH (Table 1). USH men had a 20% (SIR = 1.20, 95% CI = 1.05, 1.37) higher risk of liver cancer than men in PR. Whereas, NHW men and women had 41% and 50% lower risk of liver cancer than PR men and women, respectively. No significant differences (p ≥ 0.05) in incidence rates were observed between NHB and PR men; although NHB women showed a 29% (SIR = 0.71, 95% CI = 0.59, 0.85) lower risk of liver cancer than PR women. Mortality rates (per 100,000) from liver cancer in men ranged from 6.9 in NHW to 13.6 in PR; and in women from 3.3 in NHW to 7.8 in PR (Table 1). NHW, USH and NHB men and women had significant lower risks of death from liver cancer than men and women in PR, ranging from 14% to 58% (p < 0.05) reduction in risk. In all racial/ethnic groups, both the incidence and mortality rates of liver cancer were significantly higher among men as compared to women (Table 1).

#### Trends (1992-2003)

Although constant incidence trends (p > 0.05) were observed in men and women in PR and in most of the other racial/ethnic groups, the incidence trends of liver cancer showed significant increases among NHW men (APC = 2.0%), NHB men (4.9%) and NHW women (APC = 2.1%) (Figure 1 and Figure 2). With respect to mortality, the rates showed relatively constant trends (p > 0.05) for all racial/ethnic groups, except for NHW women who experienced an increasing mortality trend from liver cancer (APC = 0.9%).

### Cervical cancer

#### Rates (1999-2003)

The incidence rates (per 100,000) of cervical cancer ranged from 5.9 in NHW women to 12.7 in USH women. Although no significant differences in cervical cancer occurrence were observed between NHB and women in PR, USH women had 40% (SIR = 1.40, 95% CI = 1.28, 1.53) higher risk of cervical cancer than women in PR (Table 1). Whereas, NHW women had 35% lower risk of cervical cancer than women in PR (SIR = 0.65, 95% CI = 0.60, 0.70). Mortality rates (per 100,000) of cervical cancer ranged from 2.1 in NHW women to 5.1 in NHB women. No significant differences in mortality from cervical cancer were observed between women in PR and NHW. Whereas, USH women had a 41% and NHB women had a 2-fold significant higher risk of death from cervical cancer than women in PR (p < 0.05, Table 1).

#### Trends (1992-2003)

Significant decreases in the incidence trends of cervical cancer were observed for women from all racial/ethnic groups from 1992-2003, ranging from -2.2 for PR to -4.2% for USH. Similarly, significant decreases in cervical cancer mortality were also observed for all groups, except for PR where the decline was non-significant (p > 0.05) (Figure 2).

## Discussion

Our study adds updated information to the previous knowledge on infection-related cancers in PR as compared to specific racial/ethnic groups in the US [7,10,11]. The higher incidence and mortality rates of stomach, liver and cervical cancer observed among PR, NHB and USH as compared to NHW are consistent with previous studies in these populations [7,10,11,14]. Given the infectious etiology of these cancers, these results might suggest a higher prevalence of certain infectious agents among these populations, and of the risk factors associated with these infections and neoplastic conditions.

### Liver Cancer

#### Rates

Disparities in incidence rates of liver cancer among the studied racial/ethnic groups are likely the result of variations in the distribution of risk factors in these populations, such HCV/HBV infection, genetics and environmental factors or HBV vaccination coverage [27,28]. The higher risk of liver cancer in men as compared to women in PR and racial/ethnic groups studied is consistent with previous data for PR [29,30] and the US [14]. This has been explained by higher prevalence of risk factors for liver cancer in men as compared to women [31,32], such as higher alcohol consumption and HBV/HCV infection [33-35]. In the US, the prevalence of HCV in the general population ranges from 1.5% in NHW, 3.0% in NHB [19] and 2.3% in PR [34]. Similarly, the prevalence of HBV infection has also been shown to be greater for NHB than for NHW [36]. Although data on HBV and HCV for the USH population is scarce and may not accurately reflect the true proportion of people infected [33]; a higher prevalence of HBV in the majority of Latin American countries than in the US has been documented [37], but data for HCV is very limited [38]. Although a higher incidence of both HCV and/or HBV infection among USH and PR than among NHW is difficult to document, high risk behaviors for HCV infection, such as drug use and needle-sharing are high in PR and among NHB and USH [39,40]. In addition, lower HBV vaccination coverage has been documented for NHB and USH children and adults in the US [27,28,34]. Finally, of special interest are the significantly higher mortality rates from liver cancer among men and women in PR compared to USH, NHW and NHB. This result highlights health disparities between the study groups, probably explained by higher exposure to HCV/HBV, more advanced stage at disease diagnosis, decreased access to effective therapies and overall access to care in PR that warrants further attention and research.

#### Trends

Our study showed relatively constant trends in the incidence and mortality from liver cancer in PR, USH and NHB; however, incidence trends among NHB men increased. Increasing incidence and mortality patterns were also observed among NHW men and women, a result consistent with other studies in the US [41]. A possible explanation for the rising incidence of liver cancer among NHW and NHB men is an increase in the prevalence of cirrhosis, mostly secondary to chronic infection with HCV [41,42]. Also, the HCV cohort of patients infected by intravenous drug use during the 1960's-1980 has been responsible for the increasing incidence trends of liver cancer in the US [41,42]. In PR, despite the constant trends in the incidence of liver cancer, it is expected to increase in the next decades as a result of the projected higher prevalence of HCV-related cirrhosis [42]. Meanwhile, the constant mortality trend in most study groups suggests that no major advances in prevention and/or treatment of liver cancer have been achieved. For example, no treatment has been proven effective in preventing progression to chronic liver disease and HCC among HBV carriers [6].

### Stomach Cancer

#### Rates

The higher incidence of stomach cancer in PR compared to NHW (although similar to that of NHB and USH) suggests a higher prevalence of stomach cancer risk factors in this population, including *H. pylori *infection as well as other factors associated with infection, or other known risk factors for stomach cancer such as a high intake of salty foods and/or N-nitroso compounds [43-45]. Although data on the prevalence of *H. pylori *is scarce in PR, infection rates with *H. pylori *are higher in NHB (52.7%) and USH (61.6%) populations as compared to NHW (26.3%) in the US [45]. This pattern supports the higher incidence rates from stomach cancer among Hispanic populations in the US, similar to Latin American countries [2,10,13]. These results suggest a higher burden of *H. pylori *infection in PR, USH and NHB than in NHW, thus, additional data in PR is warranted. Meanwhile, the higher risk of stomach cancer in men as compared to women in PR and in all racial/ethnic groups studied is consistent with data for PR [29,30] and the US [14]. This difference is explained by a higher prevalence of risk factors for this cancer in men as compared to women [31,32], such as H. *pylori *infection [33-35]. Mortality rates from stomach cancer were also significantly higher for men and women in PR than for USH and NHW (although similar to NHB). These differences might be explained by differences in stage of disease at diagnosis, decreased access to effective therapies and overall access to care in PR and among NHB. These differences warrant further attention and research. Among prevention strategies, early eradication of *H. pylori *infection in high risk patients (such as those of patients with peptic ulcer disease) should be promoted in PR, as this reduces gastric cancer risk by reversing many biochemical, genetic and epigenetic changes in stomach cancer induced by *H. pylori *infection [46].

#### Trends

Decreasing incidence trends from stomach cancer observed in our study are consistent to patterns observed worldwide [6]. Although the reasons for these declines are not well understood, they are thought to be related to improvements in diet, food storage, as well as a reduction in the prevalence of *H. pylori *infection caused by increasing levels of living standards over the past 50 years and an increased use of antibiotics [6,47,48]. The same could be hypothesized for PR, given the improvements in living conditions, education and sanitation [49]. In addition to these factors, decreasing mortality trends may also be explained by increased access to care in all population sub-groups.

### Cervical Cancer

#### Rates

The higher incidence of cervical cancer observed among USH, NHB and PR could reflect a potential higher prevalence of HPV infection in these populations or lower screening rates in these groups. Although no population-based data for the prevalence of HPV have been reported in PR that might explain the burden of cervical cancer among women in this population, a high incidence of cervical cancer has been observed in developing Latin American countries (33.5 per 100,000) and the Caribbean (33.5 per 100,000) [50]. Although studies in the 1990's suggested a higher prevalence of HPV in USH and NHB in the US as compared to NHW [51], which support the higher burden of cervical cancer in these groups, more recent studies show a higher prevalence among NHB but not among USH [52]. The higher incidence of cervical cancer in these groups may also be influenced by the high HIV/AIDS burden in these minority populations [53,54], as cervical cancer is an important AIDS-defining illness and may be the most common AIDS-related malignancy in women [55]. Regarding mortality, PR and NHW had similar risks of death from this malignancy, while USH and NHB had increased risk. Disparities in cervical cancer screening compliance between these racial/ethnic groups could also account for some of the observed differences in both incidence and mortality. Nonetheless, the lower prevalence of cervical cancer screening in PR (70.2% in 1996, 73.3% in 2002) than in the US (84.7% in 1996, 87.2% in 2002) [35,56] and to those of the studied racial/ethnic groups (>85%) does not support the lower mortality from the disease in PR as compared to NHB and USH.

#### Trends

Decreasing patterns of cervical cancer incidence and mortality are consistent with data in PR [57] and the US [58] and supported by the increased use of cervical cancer screening in these populations. Although they could also be influenced by decreasing patterns of HPV infection in these populations, historical data of HPV infection to support this hypothesis is unavailable. Nonetheless, this is less likely given that infection with HPV is associated with high risk sexual practices, such as, early age of sexual intercourse, multiple sex partners, and lifetime number of partners; and in fact, high risk sexual practices that have increased in younger cohorts [59].

### Strengths and Limitations

This study provides updated population-based data on the burden of infections-related cancers in PR compared to racial/ethnic groups in the US. Among study limitations, incomplete information regarding stage at diagnosis of cancer cases in PR limits our ability to consider the impact of staging on cancer trends. Also, even though PR is a Hispanic population, Hispanics in the US constitute a heterogeneous group of persons from a variety of Hispanic origins that show substantial variability in cancer rates [11]. Even though the Hispanic population residing in the US described in our study is not directly comparable to the Puerto Rican population living in PR, racial/ethnic group comparisons identify disparities in the burden of disease and generate hypotheses about the role of environmental, genetic, social, and lifestyle factors on cancer occurrence [7,60,61].

## Conclusions

In conclusion, the higher overall burden of liver, stomach and cervical cancer among PR, USH and NHB, as compared to NHW, shows a health disparity between these groups that could be explained by various factors: 1) higher prevalence of infection with oncogenic infectious agents in these racial/ethnic populations, 2) lower rates of appropriate screening and early detection of cancer in these groups, and 3) differences in access to advances in preventive and treatment options among these populations. Nonetheless, the declining trends in the incidence and mortality of stomach and cervical cancer in PR might be explained by improvements in socioeconomic status, reductions in the burden of infectious agents and improvements in access to preventive and treatment care. Further research is warranted to understand the observed differences in cancer occurrence across these populations, with a particular focus on the burden of cancer-related infectious agents in these populations and the factors associated with disease progression.

## Competing interests

The authors declare that they have no competing interests.

## Authors' contributions

APO conceived the study, participated in its design and coordination and wrote the manuscript. MSS and WAC performed the statistical analysis and helped to draft the manuscript. GTL, CMP and CR helped to draft the manuscript. JP and NF provided the data and helped to draft the manuscript. ES supported the conception of the study, participated in its design and coordination and helped to draft the manuscript. All authors read and approved the final manuscript.
